# The hidden structure of consciousness

**DOI:** 10.3389/fpsyg.2024.1344033

**Published:** 2024-04-08

**Authors:** Bruno Forti

**Affiliations:** Department of Mental Health, Azienda ULSS 1 Dolomiti, Belluno, Italy

**Keywords:** explanatory gap, explanandum, hidden conscious structure, phenomenal analysis, early vision, multiple hierarchical segregation, hierarchy of spatial belongings

## Abstract

According to Loorits, if we want consciousness to be explained in terms of natural sciences, we should be able to analyze its seemingly non-structural aspects, like qualia, in structural terms. However, the studies conducted over the last three decades do not seem to be able to bridge the explanatory gap between physical phenomena and phenomenal experience. One possible way to bridge the explanatory gap is to seek the structure of consciousness within consciousness itself, through a phenomenal analysis of the qualitative aspects of experience. First, this analysis leads us to identify the explanandum concerning the simplest forms of experience not in qualia but in the unitary set of qualities found in early vision. Second, it leads us to hypothesize that consciousness is also made up of non-apparent parts, and that there exists a hidden structure of consciousness. This structure, corresponding to a simple early visual experience, is constituted by a Hierarchy of Spatial Belongings nested within each other. Each individual Spatial Belonging is formed by a primary content and a primary space. The primary content can be traced in the perceptibility of the contents we can distinguish in the phenomenal field. The primary space is responsible for the perceptibility of the content and is not perceptible in itself. However, the phenomenon I refer to as *subtraction of visibility* allows us to characterize it as *phenomenally negative*. The hierarchical relationships between Spatial Belongings can ensure the qualitative nature of components of perceptual organization, such as object, background, and detail. The hidden structure of consciousness presents aspects that are decidedly counterintuitive compared to our idea of phenomenal experience. However, on the one hand, the Hierarchy of Spatial Belongings can explain the qualities of early vision and their appearance as a unitary whole, while on the other hand, it might be more easily explicable in terms of brain organization. In other words, the hidden structure of consciousness can be considered a bridge structure which, placing itself at an intermediate level between experience and physical properties, can contribute to bridging the explanatory gap.

## Introduction

According to Loorits (2014), if we want consciousness to be explained in terms of natural sciences, we should be able to analyze its seemingly non-structural aspects, like qualia, in structural terms. During the last three decades, numerous authors have sought to identify the structure of Phenomenal Consciousness (PC) in classic neuronal organization (Crick and Koch, 1998; Dehaene et al., 1998; Tononi and Koch, 2008; Jerath and Crawford, 2014; Calabrò et al., 2015; Koch et al., 2016; Boly et al., 2017; Gallotto et al., 2017; Polák and Marvan, 2018; Noel et al., 2019; Maillé and Lynn, 2020; Seth and Bayne, 2022), in the activity of electromagnetic fields (McFadden, 2020, 2023; Ward and Guevara, 2022; Jones and Hunt, 2023), or in quantum physics (Hameroff and Penrose, 2014; Tuszynski, 2020). However, all these studies do not seem to be able to bridge the explanatory gap (Levine, 1983) between physical phenomena and phenomenal experience (Marius, 2014; Skokowski, 2022; Jones and Hunt, 2023; Sanfey, 2023).

Many theories have addressed non-specific aspects of consciousness, such as access-consciousness (Block, 1995, 2005; Baars, 2002; Tyler, 2020), meta-representation (Gennaro, 2004; Brown et al., 2019), global access (Dehaene et al., 1998; Dehaene, 2014), unity (Bayne, 2010), integration, (Tononi, 2008; Tononi and Koch, 2015; Brogaard et al., 2021; Hirschhorn et al., 2021), intentionality (Crane, 2003, 2009), selection (Zeman, 2001; Schwarzkopf and Rees, 2015). In the absence of specific features of consciousness, there is a risk of formulating a theory that refers to something that is compatible with the absence of consciousness. The specific characteristics of consciousness can be attributed to its phenomenal aspect, which are usually traced back to qualia (Dennett, 1988; Searle, 1997) and what it is like to be in a certain state (Nagel, 1974).

I think that correlating phenomenal experience with certain aspects of neuronal processes – even discovering the proper level of organization of the neural activity (Revonsuo, 2006) – is not enough to bridge the explanatory gap and thus solve the hard problem. In my opinion, the nature of the brain structure is such that it cannot explain – at least directly – experience. All the data we have thus far – and probably also those we might have – seem to indicate that a brain in the broad computational sense is unable to account for experience (Toribio, 1993). Phenomenal experience and brain structure are too different or “distant” to be directly compatible. This difference is probably the basis for the very conception of the explanatory gap and the formulation of the hard problem. On the contrary, structural aspects of consciousness can be found in phenomenal experience. Consequently, a possible alternative is to look for the structure of seemingly non-structural aspects of consciousness (Loorits, 2014) not in the neuronal substrate, but in consciousness itself, through a phenomenal analysis of the qualitative aspects of experience that starts from its simplest forms.

An essential premise is to reformulate the explanandum of consciousness. In fact, qualia do not have a phenomenal existence as isolated entities. Furthermore, the qualitative aspects usually analyzed in the literature - such as the redness of red or the painfulness of pain – must be placed in a more complex structural context than is commonly believed. The simplest qualitative aspects – such as those related to being an object, background or detail - can be found in early vision. They are involved in perceptual organization and necessarily have relational significance. Such phenomenal qualities, which are manifold and different from each other, are perceived in relation to each other and seem to form a unitary whole. We can say that the explanandum of consciousness is a unitary set of qualities, i.e., a set of qualities closely dependent on each other, which we can find in its simplest forms in early vision (Forti, 2024). Of course, unity *per se* is not specific to consciousness. However, in this case unity concerns specific properties of consciousness such as the qualitative aspects. The co-presence of the qualitative aspect and the unity aspect is thus crucial in identifying the explanandum of consciousness.

Although early vision is characterized by interdependent qualitative components that form a unitary whole, we cannot find in it the structure of seemingly non-structural aspects of consciousness. Phenomenal appearance alone does not seem sufficient to identify a unitary structure of consciousness. However, the closeness of these characteristics to a unitary structure prompts us to delve into a less explored territory, using the components of experience also as possible explanans. I hypothesize that the structure of consciousness can be found in consciousness itself on the basis of the possibility that the aspects we attribute to Phenomenal Consciousness (PC), in addition to being explananda – whereby we wonder how subjective experience, made up of qualia, sensations and feelings, emerges or is produced by brain activity – may contribute to an explanation of consciousness itself.

A not insignificant consequence of considering the phenomenal aspects of consciousness *only* as explananda is that, in almost all theoretical approaches, the analysis of these aspects is inadequate. When one merely explains the non-specific aspects of consciousness, one does not perform a phenomenal analysis at all. The phenomenal aspects are simply pushed aside or ignored. In other cases, the phenomenal analysis is very sketchy, limited to only a few elementary aspects like the redness of red and the painfulness of pain. From this point of view, the need to restrict and simplify as much as possible what we have to explain is understandable. However, these approaches lead to ignoring the relational aspects of so-called qualia and to underestimating the richness of the internal structure of experience, even in its simplest forms, and thus to an unrealistic view of the experience that one wants to explain. Phenomenologists have highlighted this issue well: “we will not get very far in giving a scientific account of the relationship between consciousness and the brain unless we have a clear conception of what it is that we are trying to relate. To put it another way, any assessment of the possibility of reducing consciousness to neuronal structures and any appraisal of whether a naturalization of consciousness is possible will require a detailed analysis and description of the experiential aspects of consciousness” (Gallagher and Zahavi, 2008). My approach goes beyond the understandable need to better define the explanandum. The possibility that the phenomenal aspects of consciousness may also be useful elements in identifying an explanation prompts us to analyze them carefully and in detail, taking an interest even in secondary or seemingly insignificant phenomenal aspects.

As I will explain in the next sections, I postulate the existence of *non-apparent* parts of experience and hypothesize that consciousness possesses a *hidden* structure, one that comprises both apparent and non-apparent constituents. I call it the Hierarchy of Spatial Belongings (HSB). This structure can explain the unity of early visual experience and its main qualitative aspects, i.e., its being a unitary set of qualities. At the same time, it better lends itself to being correlated with certain physical processes, helping to bridge the gap between experience and brain processes.

The reasons for taking this hypothesis into consideration arise from the analysis of generally neglected phenomenal aspects such as surroundedness and overlapping of the contents of the field. Another element that suggests the possibility that in consciousness we can find elements that can help explain consciousness itself is the problem of appearance. I hypothesize that appearance depends on something which could be responsible for making it appear, but which would not have in itself the property of appearing. Therefore, what appears would be a clue to the existence of what does not appear. This something could belong to the region surrounding the object, to which we attribute a phenomenal nature of background or space.

## The multiple hierarchical segregation of the perceptual field

The mechanism underlying a hidden structure of consciousness can be identified in the model of Multiple Hierarchical Segregation (MHS) of the perceptual field, which I presented in detail in another article (Forti, 2015). MHS is an alternative model of perceptual organization to the Gestalt model. A limit of Gestalt theory is the lack of a comprehensive view of perceptual organization. This can be seen in the distinction between figure-ground segregation and grouping, as well as in the proliferation of principles of grouping (Wagemans et al., 2012). The traditional view is that, once the object is identified (Pinna, 2012), grouping takes place among the components of the field which have an object nature through a heterogeneous set of field organization principles. I have proposed a mode of organization in which the spaces of the field play an active role. This model provides a simpler explanation than the traditional principles and is compatible with a unitary structure of the visual field.

The conditions under which we see a simple figure can be derived from the nature of the figure and of the ground. The perception of a black triangle is conditioned by the brightness of the background. The bigger the object-foreground difference is, the more vivid our conscious experience will be. Therefore, these conditions imply the division of a field into two homogeneous regions, one internal to the other and the two contrasting with each other (Todorović, 2008; Wagemans et al., 2012). One may say that the conditions under which we see a simple shape on a homogeneous background consist in the *Surrounding Contrast (SC)* of the structure of the proximal stimulus. All else being equal, figures in which the SC of the proximal stimulus structure is strongest will tend to visually dominate the others. The strongest contrast of the proximal stimulus is the one in which there is the greatest difference in the response of the receptors to two concentric regions of the stimulus field.

According to the MHS model, there is a correlation between the SC gradient of the structure of the proximal stimulus and the progressive segregation of the perceptual field. There is a SC when a spatially extended region of the proximal stimulus contrasts or is inhomogeneous with the whole surrounding region. These conditions can occur, albeit with some differences, in several perceptual modalities. Since all relationships involve the field of the stimulus in its entirety, we have to imagine that several SCs of the structure of the stimulus are overlapping in a complex way.

**Figure 1:**
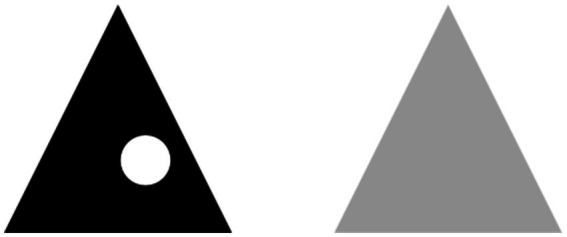
A simple example of multiple hierarchical segregation.

The fact that all relationships involve the field of the stimulus in its entirety does not occur at a phenomenal level. In fact, it seems to occur only for the main object. As can be seen in Figure 1, the strongest SC corresponding to the black triangle not only causes it to phenomenally prevail over the other elements, but it also brings about a subdivision of the field into two asymmetric areas which we perceive as figure and ground. Unlike the main segregation, the other segregations which derive from the smaller SCs do not seem to affect the field in its entirety, but the areas which formed as a result of the first segregation. We see the gray triangle *in the region which acts as a background* to the black triangle, i.e., inside a space which does not include the whole framed area, but only the white space surrounding the black triangle, and we see the small white circle *inside* the black triangle.

Similarly, a face segregates from the background and it is in its turn affected by a process of segregation. This process does not affect the whole field, but only the object, i.e., the face which acts as a “background” to the eyes, nose and mouth. The pair of eyes segregates from the face; in its turn, each eye segregates from the region occupied by the pair of eyes. This process appears as the most appropriate explanation of what occurs when we perceive objects such as a house or a tree, which are internally complex and which are perceived in a context which is in its turn internally complex.

**Figure 2:**
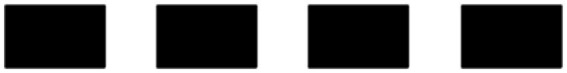
A broken line.

As in the pair of eyes, the progressive segregation of the field occurs not only when an object is located inside another. The term SC refers to a region located inside the field, without necessarily identifying it with a continuously contoured figure, and it can correspond to the grouping of several objects into a gestalt. A broken line (Figure 2) perceptually appears as prevalent because, despite the discontinuity of the parts which form it, the line corresponds to the strongest SC of the structure of the stimulus. The SC corresponding to the individual dashes is smaller than the SC of the line due to the presence of the other dashes in the external space, while the individual dashes have more or less an equivalent SC. As a consequence, the region corresponding to the line is secondarily subdivided into the four dashes and we see a broken line, i.e., *a line made up of dashes*. Like the white circle belongs to the black triangle, the dashes belong to the line – and not to the whole image. Each dash, despite not prevailing over the others, is seen “against the background” of a region which includes the other dashes. In general, we can say that the simple rule stating that, given a visual field, the perceived object corresponds to the strongest SC of the proximal stimulus accounts for several aspects of perceptual organization, thus unifying Gestalt laws. My previous article (Forti, 2015) provides a detailed description of the phenomena that are usually explained on the basis of grouping principles.

In short, there is no grouping of the perceived dashes on the basis of their similarity and proximity, as stated in Gestalt laws. Instead, there is a progressive segregation of the structure of the stimulus, i.e., a process of MHS. MHS is correlated with the SC gradient of the proximal stimulus. The term progressive should not be interpreted in a temporal sense, but in a hierarchical sense. The segregation which determines the perception of the line is hierarchically superior, while the segregations which determine the perception of the dashes are subordinate to it.

It is evident that the perceptual situations selected by gestaltists for their analyses favor the possibility of “seeing” groupings of elements instead of the progressive segregation internal to the field, as is the case when observing the most common perceptual situations. However, the progressive segregation internal to the field can be “seen” also in the perceptual situations analyzed by gestaltists. In this perspective, what gestaltists call grouping by proximity and by similarity can be considered a sort of atypical MHS. A broken line is a sort of incomplete segregation, because it includes both what we attribute to matter and what we attribute to space. Nonetheless, this region tends to segregate anyway in the presence of a sufficient SC of the stimulus. Segregation is atypical in that an incomplete segregation such as the one of the broken line, arising from a stronger SC of the stimulus, prevails over the complete segregations of the individual dashes.

According to Searle (2004), there are two aspects to the Gestalt structure of consciousness: (1) the capacity of the brain to organize perceptions into coherent wholes; (2) the capacity of the brain to discriminate figures from backgrounds. Similarly, Wagemans et al. (2012) state that “perceptual grouping and figure-ground organization, although intimately connected, are not the same process.” If instead we think of the phenomenal field as a hierarchy of relationships which form following the progressive segregation of the field, these two aspects can be unified.

The MHS model seems consistent with both the possibility of a single mode of field organization and the need to account for the progressively less significant aspects of phenomenal experience. The main relationship concerns the whole field, the less important relationships concern the parts that formed as a result of the first subdivision, and so on. Unity seems to derive not so much from the existence of elements of homogeneity and coherence in the perceptual Gestalt as from the internal subdivision of the conscious field. As a result of the SC gradient of the proximal stimulus, the visual field is gradually segmented within itself, and each subdivision appears to be strictly dependent on the others. This seems to account for the unity of the conscious structure as well.

However, the progressive subdivision of the field within itself highlights a shortcoming of the MHS model. It is the fact that, while appearing homogeneous, several parts of the field would at the same time be composite. The object is contained within a background but, at the same time, it contains details within itself. The background contains the object within itself but, at the same time, it is contained within an additional background. The problem affects most regions of the field, even under the simplest perceptual conditions. The white space inside the box in Figure 1 can have four perceptual properties: it acts as the background of the black triangle, it is part of a larger background, it acts as the background of the gray triangle, and finally – being a box – it is an “object” seen against the background of the external space. If object and background are to some extent composite, what are they composed of? Since we do not see a background superimposed on a figure or, respectively, a figure superimposed on a background, how can we reconcile the background role of the space inside the box with its role as an “object” seen against the background of the space outside the box?

Other authors have also highlighted this problem. According to Peterson and Salvagio (2010), a region can be a ground along some portion of its bounding edges, and a figure along other portions. Even though the white background in Figure 1 is unshaped near the border it shares with the smaller black region, it is shaped by the outline border it shares with the larger surrounding white region. But the subdivision of this region into two juxtaposed parts seems artificial, especially in the case of small backgrounds. Moreover, it is not compatible with further subdivisions of the field. Another way to deal with this problem is to assume that we see these different aspects of a field region at later times (Searle, 2004). In fact, we do not necessarily separate – at least sharply – a region into a part that we liken to an object and a part that we liken to a background, nor do we see the different parts of an image one after the other. This means that the internal organization of the field involves the simultaneous presence of values whose nature we struggle to understand. Is the MHS hypothesis therefore wrong in that it is phenomenally untenable?

## The hierarchy of spatial belongings

The MHS model solves the problem of the unity of conscious structure. However, it poses the problem of the composite nature of many regions of the field that we consider homogeneous. A relatively simple solution is that the only difference between the outcomes of the individual segregations is their hierarchical value. The question can be posed in the following way: if we expect a multiplicity of relations which hierarchically overlap each other as a result of the progressive segregation of the field and make us see how we see what we see, what is the nature of the outcome of each segregation?

I propose that the single segregation of the field would not lead to the formation of figure and ground, so we cannot speak of figure-ground segregation. I call Spatial Belonging (SB) the “simple” relationship, a kind of proto-image, produced by each segregation. I use this definition because belonging to a space is a *sine qua non* for any content to be conscious, for it to be perceived. A SB consists of a primary content and of a primary space. They are content and space in the absolute sense of the term if they do not overlap with other contents or spaces.

What are the properties of the two concentric regions of the SB? I propose that the primary space has the property of allowing the primary content to appear, or to be perceived, and that this occurs through a relationship between contrasting outer and inner regions. This means that a content cannot be perceived unless it is surrounded by a primary space and that this space, while making it appear, is not perceivable. All spatial belongings are characterized by these properties. The difference is that Spatial Belongins arising from a stronger SC prevail over the others and contain them within themselves. It should be pointed out that we do not experience content perceptibility at the level of the individual SB, which we cannot access, but at the phenomenal level. While primary content and primary space are at a level we can call sub-phenomenal, our perceptual experience is made up of overlapping Spatial Belongings nested within each other. Since primary space is not perceptible, it remains “hidden” from our experience. However, it is not phenomenologically inert. I will address this issue in the section “Appearance.”

**Figure 3:**
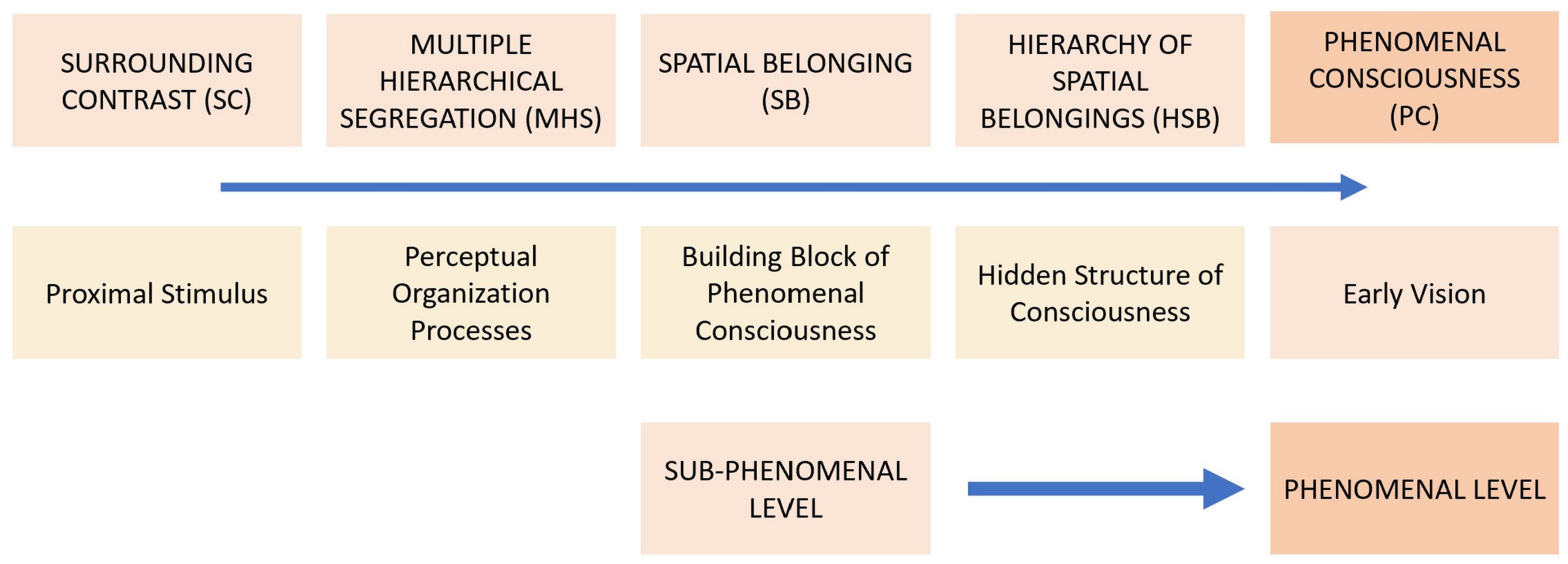
Starting from the SC of the proximal stimulus, the processes of MHS produce the hierarchical organization of SBs. This hidden conscious structure (HSB) corresponds to the phenomenal level of early vision (PC).

Are primary content and primary space consistent with our experience? Can we reconstruct the phenomenal level of early perception from Spatial Belongings? Spatial Belongings are the building blocks with which early vision is constructed in its qualitative and structural aspects. According to the MHS model, the total field segregates into two concentric regions forming the main SB. In the presence of inhomogeneity, each of the two regions thus formed segregates within itself in turn, resulting in smaller Spatial Belongings. Further segregations result in progressively smaller and less phenomenally relevant Spatial Belongings, until fading. MHS causes different Spatial Belongings to largely overlap with each other. For example, the primary content of the main SB in Figure 1, which includes the area of the black triangle together with the white circle, overlaps with the SB which includes the area of the black triangle as primary space and the area of the white circle as primary content. We could say that most of the field regions we perceive, including the seemingly simplest ones, are at least both primary content and primary perceptual space as a result of the – multiple – overlapping of Spatial Belongings in a hierarchical structure which I call the Hierarchy of Spatial Belongings (HSB). Essentially, the Multiple Segregation of the perceptual field determines the individual Spatial Belongings and their hierarchical organization, accounting for the phenomenal nature of early perception. What we see is the effect of the relationship between overlapping regions nested within each other. The HSB corresponds to early vision but, unlike the latter, it cannot be experienced as such because of the presence of hidden components in it (Figure 3).

Primary contents ensure the perceptibility of what we can call phenomenal contents. Of the many terms used to describe what we perceive, the term ‘content’ appears to be the most generic. Etymologically, content is necessarily inside something. Phenomenally, and thus not as primary content, a content is anything that we can perceive in a phenomenal field and that appears sufficiently separate from other parts of the field. It is the thing on the basis of which we can make a phenomenal distinction. Despite their differences, all regions of the field have this property in common. From this point of view, a background is also a content.

Phenomenal contents coexist in the perceptual field, not only juxtaposed next each other, but also overlapping each other. In Figure 1, the black triangle, the white circle, and the object consisting of the black triangle with the white circle on the inside are all contents. Both the broken line and the dashes that make it up are contents. If a scented-red-rose is a content of our experience, so are its shape, its scent and its red color, since, while belonging to the overall gestalt of the rose, they are sufficiently separate that they can be perceived as contents. Similarly, the word ʻrose’ is a content as are the individual letters that make it up.

Also the phenomenal nature of the object and of the background derives from the overlapping of the content and space components. But what differentiates the object from the background if both are formed by the overlapping of primary content and space? My proposition is that we perceive a region of the field as background when the role of the space to phenomenally define prevails over the role of the content to be defined. In the main background, the predominance of the space component is due to the fact that it is part of the main SB as a result of its stronger SC. The background has, at the very least, a dual nature: of content, on the basis of which it can be perceived; and of space, which makes us perceive the object it defines. The background is perceived through an external space, but its predominant component is to make us perceive the content it bounds, so it is perceived as *empty space surrounding the figure*.

If we keep in mind that a visual object differs from a simple shape because of its constitutive inhomogeneity, the space component is also present in the perception of the object. We perceive an object when the role of the content to be defined prevails over the role of the space to define. This is why we do not see the black portion of the main figure in Figure 1 as a background. However, a share of the attention we pay to the black triangle is subtracted from it to focus on the white circle it contains. The difference between background and object is that the component of phenomenally defining prevails in the former, while it is of lesser importance in the latter. This means that in the object the perception of the component attributable to the primary perceptual space is more difficult than in the background, although it is unquestionably present. It corresponds to the phenomenal datum whereby we see the white circle on the inside as a detail.

It should also be noted that object and background, in their content and space components respectively, represent the outcome of the main segregation. The intertwining of content and space does not account only for the phenomenal characteristics of object and background. Because of the additional subdivisions that occur within it, the external space defined by the main segregation is more complex than the generic notion of background might suggest. The secondary object, i.e., the grey triangle, is both content and part of the background, so it is seen against the background of the main object and at the same time is part of it. Moreover, the background of the main object, especially in the immediate surroundings, tends to converge on the secondary object.

Also to understand the nature of a detail it is necessary to take into account the relationships between the Spatial Belongings of the perceptual field. The Spatial Belonging to which the black triangle belongs as content together with the white circle prevails over the Spatial Belonging overlapping with this content, which includes the black triangle as space and the white circle as content. The SB between the black triangle together with the white circle and the overall surrounding space is the main one as a result of the stronger SC of the proximal stimulus. This is the reason why the black triangle is perceived as an object and not as a background and the white circle is perceived as a detail of the triangle and not as a phenomenal object in the full sense of the term. It is both content and part of a larger content that prevails over the former. Because of the limitations of the paper, I will limit myself to analyzing these features of early vision.

The variability of the relationships involved also accounts for all the intermediate situations and varying degrees of prevalence of content over space or vice versa, including the gradual way in which we move from object to background. If we look at the pen lying on a book, the book is more than a background. It may become more important and even become an object within which we recognize the detail of the pen.

Finally, the idea of a HSB appears compatible with the progressive fading of the phenomenal field. This neither means perceiving the entire field equally, nor making experience coincide with focused consciousness alone. The notion of HSB implies that there is a progressive fading of perceptibility from the main content to the contents that are gradually subordinated to it. If we consider the field as a whole, the parts we perceive in relation to others are progressively *fading*, especially – but not only, if we think of change blindness (Noë et al., 2000) – toward the outside of the field. This gradualness is entirely compatible with the richness that characterizes all our phenomenal experience. It is true that in change blindness we cannot see the changes that affect certain parts of the field, so much so that some believe that this phenomenon would demonstrate that we see much less in the perceptual field than we think (Rensink, 2004; Scrivener et al., 2021). However, it is also true that change blindness is based on perceptual situations in which *dozens* of spatial belongings are formed. It is worth noting that in very simple stimulus conditions, as in many of those studied by gestaltists, we can sufficiently perceive all the relations in the field.

Of course, it is well known that consciousness is made up of parts that we can see well and of parts that we can see less well. However, MHS allows us to explain the gradualness of this phenomenon and the structural relationship between focus and fading. The perceptual field is characterized by multiple relationships of surroundedness, the Spatial Belongings, which gradually decline from what we perceive distinctly to what we perceive with increasing difficulty, until gradual disappearance from the phenomenal field. At the same time, the less significant relationships of surroundedness depend on the more significant ones and occur within the subdivisions of the field generated by the latter. In other words, the individual Spatial Belongings are nested to each other on the basis of a hierarchical organization. With its hidden components, the HSB is the structure underlying the perceived unity of the visual field, even in situations where the contents of a scene seem to be arranged randomly. Any element is part of the whole as an outcome of the progressive subdivision of the perceptual field.

## Surroundedness

The hypothesis I put forward is based on the analysis of two generally neglected relationship modes present in the perceptual field. The first is the belonging of contents to a space, or surroundedness, and the second is the overlapping of the contents of the field.

One of the difficulties in understanding consciousness stems from the fact that the background and the fringe aspects are underestimated, as James (1890) pointed out with his metaphor of the pails in the river. However, this metaphor should be applied not only to the flowing water of a river, but also to the still water of a lake, because in this regard the important relationships are spatial as well as temporal.

Despite the fact that the relationship between foreground and background has often been included among the properties of consciousness (James, 1890; Zeman, 2001; Edelman, 2003; Searle, 2004; Northoff et al., 2023), many approaches have tended to make consciousness coincide with contents (Schulte, 2023) and focused consciousness, neglecting unfocused aspects. Of course, I do not intend to claim that the background is different from what it appears. The background is phenomenally less significant than the content. The problem is that, taking its phenomenal significance as a starting point, it is considered at best an ancillary element, which accompanies the content. Some authors have considered the background, along with fringe aspects, as degenerate information, or even as something that deceives and misleads us (Dennett, 1991, 2005, 2015; Noë and O’Reagan., 2000; Rensink, 2004; Prinz, 2018). The fact that the background is phenomenally less important does not mean that its role is necessarily negligible. Of course, the background is relational by its very nature. It implies the existence of a relationship with a figure, an object, a foreground. Consequently, what is underestimated is the relationship between figure and background.

The relationship between figure and background concerns not only well-defined shapes. For a visual stimulus to be perceived, a fine-grained representation is not necessary. Let us think of the perception of an indistinct spot. The ability to perceive it depends more on the contrast between content and background than on its definition. The fact that a piece of writing is blurred to the point that it cannot be recognized does not prevent such content from being consciously perceived.

Moreover, the presence of the background is essential not only in vision. Smelling a smell or hearing a sound are considered elementary conscious experiences. But even these contents are invariably perceived against the background of something. Just as we see an indistinct spot against the background of the surrounding visual space, a sound is perceived against the background of the auditory space,^1^ and we feel pain against the background of the leg. Coming from a region of the perceived space, many elementary sensations have phenomenal characteristics not unlike those of a blurred image. Even in cases where a sensation seems to occupy the entire visual field, such as when we close our eyes to experience darkness, we cannot help but experience our body. If we focus on the visual experience, our body will act as a background to the darkness we perceive and will in turn be perceived in the background of the perceptual space in which our body is located (Jerath et al., 2015). Perception is, *ab initio*, multisensory (Bennett and Hill, 2014; Bayne and Spence, 2015; O’Callaghan, 2015).

Another limitation of the classical approach to perceptual organization is that it almost exclusively analyzes the relationship between the figure and the background – in fact the main figure and the main background. First, there may be a number of backgrounds in a field. Second, it is better if we consider a relationship type like *surroundedness*. I define it as a relationship whereby a region is surrounded by or surrounds a contrasting region. It is a form of juxtaposition that occurs between two contiguous and concentric regions. Surroundedness has a broader meaning than the one we attach to the figure-background relationship. For example, it also applies to the relationship between object and detail or the relationship between primary space and primary content (SB).

The SB is the fundamental surroundedness relationship and it is indispensable for consciousness to exist. Being a relationship between a primary space and a primary content, it is a hidden relationship. It makes it possible to perceive the contents of the perceptual field and it is the basis of all phenomenal surroundedness relationships.

Phenomenal surroundedness does not concern only the background. We can see an object on a table, which has the nature of an object and is seen against the background of the floor. Moreover, in addition to *being surrounded*, the parts of the field can *surround* other parts, such as the dots on the faces of a dice, or such as the eyes and mouth of a person’s face. In this case, the relationship is reversed, in the sense that the main element is the object. However, an object is also the “space” in which its details are situated. Surroundedness does not concern only the relationship between the object and its internal components. Even a gestalt can be considered a set of elements *contained within* the region it occupies. We conceive of a gestalt as a group of elements. But, in fact, we perceive a broken line as a salient region containing spaces that interrupt the continuity of the line.

**Figure 4:**
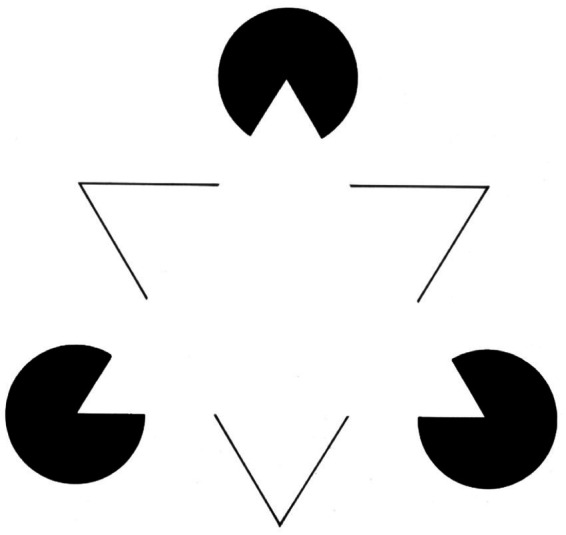
Kanizsa’s triangle.

It could be argued that first we see an object against a background that includes the other elements of the field, and that later we shift our attention to other aspects of the field. According to Searle (2004), “I see the pen against the background of the book, the book against the background of the desk, the desk against the background of the floor, and the floor against the rest of the room, until I reach the horizon of my entire perceptual field.” Based on a widespread view, this description implies that, by gradually broadening our focus, we *first* see the pen against the background of the book, *then* the book against the background of the desk, and *then* the desk against the background of the floor. In fact, if we focus our attention on the book, we see the book – on which lies a pen – against the background of the table *and simultaneously* the table against the background of the floor. While it is true that we see less well as we move away from the object on which we focus our attention, we cannot even say that we see only what we focus on *and nothing else*. This would be not only a simplistic conception of our experience. Failure to perceive the secondary parts would alter or prevent the perception of the main content. This is evident in illusory figures. We can only perceive an illusory triangle (Figure 4) if we superimpose it on three black disks and a white triangle with a black outline, which in their turn stand out against the surrounding white background. This phenomenon is even more evident in other much more common perceptual situations, such as the ones analyzed in Gestalt psychology. We cannot see a broken line without seeing – at the same time – the dashes that make it up.

## Overlapping

Another type of relationship, which is also overlooked, involves overlapping between the components of the field.^2^ While evident in some cases, phenomenal overlapping is hardly analyzed for its role in the phenomenal structure (Jerath et al., 2019). I think that overlapping in the conscious field is not only more frequent than we think, but also that it is a fundamental component of conscious organization, without which PC would not be possible.

Overlapping occurs in a number of circumstances, such as in the case of occlusions, when one object extends behind another and through amodal perception we tend to see its shape (Briscoe, 2011; Calabi, 2013; Nanay, 2018). Another well-known case of overlapping, i.e., the one of the background, which tends to perceptually extend behind the object, is even one of the basic properties of visual perception (Todorović, 2008). In fact, overlapping by occlusion is the only one that is taken seriously in the literature.

But phenomenal overlapping does not only occur when visual regions overlap with each other. The characteristics of reality in which we are interested are also auditory, tactile, olfactory and so on. Consequently, multisensory stimuli, such as observing a person talking to us or smelling the scent of a flower we admire while holding it in our hands, come to us simultaneously from the same region of space. This is reflected in our perceptions, which seem to be formed by overlapping sub-images. Overlapping also occurs in the case of different sub-modalities, for example, between form and color. With regard to the property of composition, Tononi and Koch (2015) mention the example of the perception of the blue book, but without explicitly addressing the structural function that the relationship between the two phenomenal distinctions might have at the phenomenal level.

Moreover, as we saw above, contents that can be referred to a part can overlap with contents that can be referred to the whole. Of course, overlapping can also occur with states originating internally, such as emotions, memories, thought processes or simulations (Smit et al., 2023), which I am not going to address in this paper. These few examples highlight a phenomenal reality that can hardly be disputed. However, overlapping is rarely considered a defining characteristic of phenomenal consciousness (Fingelkurts et al., 2009).

The problem of binding is to inquire how the brain binds together multiple multimodal characteristics into the unitary experience of the object, i.e., into what we conceive of as a single conscious “image” (Feldman, 2013). I am not going to delve into the role of the neuronal processes involved (Crick and Coch, 1990; Llinas et al., 1994; Revonsuo and Newman, 1999; Deroy et al., 2014; Walling, 2019), about which there is no consensus and no entirely satisfactory explanation (Isbister et al., 2018; Jerath and Beveridge, 2019; Kesserwani, 2020). But it is worth considering the possibility that binding may *also* occur at the conscious level and that it may occur through the overlapping of a number of sub-images.

The above leads us to think that overlapping is involved in the qualitative aspects usually attributable to qualia. A qualitative sensation is something that overlaps with a region of a perceived image – usually an object – characterizing it and modifying the experience (Jerath et al., 2019). By binding a certain characteristic to an object, overlapping is the way in which the unity of the object is achieved at the conscious level. Thus, the concept of overlapping allows us to place qualitative features in the context of the relations existing in the field and to assign them a structural role.

A qualitative sensation can overlap with an object, as in a yellow triangle or in the taste, pleasantness, color, and cold feeling of pistachio ice cream. Therefore, it is perceived through overlapping with an object which in turn belongs to a background, and thus through secondary or indirect belonging to the background. Or a quality may itself determine the extent of a certain region. In this case, it acquires an object-like function, thus defining a region which in turn belongs to a background.

One might counter that yellow is identified with a certain region. It is true that qualitative aspects define the characteristics of an object that has a certain form and that they can take on the form of the objects they overlap with, as in the case of color. However, qualitative aspects are independent of form, so quality – unlike an object – does not have a form of its own. In conscious perception a color may or may not extend like the object, or it may itself be the object. When yellow overlaps with a definite form such as a banana, we can tell if the yellow color has the same extent as the banana, so seeing a yellow object means seeing it uniformly yellow. Or we can tell if the extent of the yellow color is different from the banana, so we can detect streaks and their actual shape. In the case of a drawing, we can tell if the banana is colored well and, if not, we can identify the form of the color. In the case of a badly colored object, we will say that the extent of the yellow color does not perfectly match the object, but we will not attribute that form to its being yellow. We will attribute it to the region in which the color is located and which is bounded by the surrounding space through contrast. If we see that form on its own, like a spot of color, we will attribute it to the spot, not to the color itself. Even if a rainbow is made “only” of color, at a phenomenal level it characterizes itself as a *colored arc*.

In the case of a tactile or olfactory sensation, such as pain in a knee or a sound from a certain area in the external space, there are no sharp contours. The lack of a definite form seems to depend on the poor degree of definition of pain or sound. As sensations, they help create a kind of formless object, although more or less extended and located in space. In fact, even in this case pain delimits a region of the leg. It is the knee that hurts. We distinguish pain from the region of the leg that hurts, although the latter is defined by the pain itself. Similarly, a sound comes from a region of the visual landscape and presumably from something located in it.

The above analysis highlights that the qualitative aspects usually analyzed in the literature are placed in a more complex structural context than those related to being an object, background or detail. But overlapping occurs also in the simplest, seemingly homogeneous parts. Admitting the existence of multiple segregations poses the problem of overlapping not only between distinct regions of the field – such as between occluding and occluded object or between multimodal sub-images – but also within the same region of the field. MHS leads us to hypothesize that individual segregations result in a kind of proto-images, or SB, that overlap with each other.

HSB is based on the combined role of overlapping and surroundedness. Overlapping and surroundedness are constitutive of consciousness, even in the simplest forms of perceptual experience. What appear to us as juxtaposed components of the perceptual field are actually Spatial Belongings. Their spatial component is not apparent *per se* and they partially overlap with and are nested within each other.

## Appearance

Paradoxically, one aspect that can help identify *non-apparent* parts of consciousness is precisely that relating to appearance. Like the relationships of surroundedness and overlapping, it is another fundamental yet neglected aspect. We usually consider it a priority to explain the qualitative aspects of consciousness, but its appearance is something even more fundamental and such that it underlies the qualitative aspects. Aspects related to appearance should be distinguished from strictly phenomenal aspects. According to Nagel (1974), a being is conscious just if there is “something that it is like” to be that creature, i.e., some subjective way the world seems or appears from the creature’s mental or experiential point of view (Van Gulick, 2022). The problem lies not only in the way the world appears to us, in the effect the world has on us in its appearance, but also in the mere fact of appearing (Revonsuo, 2006; Whiting, 2016; Merlo, 2020).

In the simplest sense, appearance, which is nothing else than the etymological meaning of consciousness as a phenomenal entity, implies the possibility of something being perceived consciously. This can mean several things: being conscious rather than not being conscious; seeing rather than being blind, despite having other sensory experiences; distinguishing two neighboring points rather than not distinguishing them. In metaphorical terms, if on the inside of Chalmers’ zombies all is dark because they have no experience, appearance is that thing that occurs when the “light” of consciousness comes on (Baars, 1997, 2005). This idea is often associated with something magical and inexplicable. As Thomas Huxley states: “How it is that anything so remarkable as a state of consciousness comes about as a result of irritating nervous tissue, is just as unaccountable as the appearance of the djinn when Aladdin rubbed his lamp in the story.” The notion of global access in the Global Neuronal Workspace theory (Dehaene, 2014; Mashour et al., 2020), linked to brain “ignition,” is not that far from this conception and could be considered an updated version of the idea of “conscious light” ignition.

However, at least in a relative sense, appearance is something that is not evenly distributed throughout the conscious field, but it concerns some regions of the conscious field to a greater extent than others. Moreover, the latter seem somehow necessary for perception to occur. “A figure on a background … is the very definition of the phenomenon of perception, that without which a phenomenon cannot be said to be perception at all. The perceptual ‘something’ is always in the middle of something else, it always forms part of a ‘field’” (Merleau-Ponty, 1945). From this point of view, one could say that, since the relationship between object and background involves the existence of contrast, the conditions for the emergence of an elementary form of phenomenal experience do not depend on the – metaphorical – coming on of the light of consciousness, but on the development of a certain kind of relationship between darkness and light. Light certainly illuminates an object in darkness, but darkness also makes light visible. Total darkness, as well as total light, caused by the absence of a contrast between the object and what surrounds it, cannot ever constitute the totality of consciousness, as suggested by the Ganzfeld effect (Schmidt et al., 2020).

According to James (1890), one of the main characteristics of consciousness is that “it is *always* interested more in one part of its object than in another, and it welcomes and rejects, that is, chooses, all the time it is thinking.” This phenomenon is not necessarily related to attention (Pitts et al., 2018). It is not so in the case of the perception of a simple figure against the background of something (Kimchi, 2009). James states that “we find it quite impossible to disperse our attention impartially over a number of impressions.” In other words, consciousness cannot help but function in this way, so this characteristic is constitutive of consciousness itself. But even before choosing between different contents, whichever way we want to conceive of them – objects, impressions or otherwise – we choose between content and container. Consequently, if we think of the perception of a simple figure, this characteristic of consciousness might imply the very possibility of perceiving.

As we have seen above, appearance derives from SB, a hidden surroundedness relationship. Primary space does not have the property of appearing, but it rather has the property of allowing the primary content to appear. However, as we will see later in the text, it can be traced in the region surrounding any conscious content, to which we often attribute a phenomenal nature of background or space (Forti, 2009). Of course, what is figure and what is background depends on the mutual arrangement of the field regions and it may change over time. According to this hypothesis, in bistable figures we see one figure at a time because the figure is seen thanks to the surrounding region, which thus cannot be seen at the same time. Naturally, bistable perception may depend on attention and neural oscillations (Doesburg et al., 2009; Dieter et al., 2016; Davidson et al., 2018; Zhu et al., 2022).

Is there any evidence for which we can say that the space surrounding an object “makes us see” the object, while remaining unseen, and that it can be traced in PC? Preliminarly, if we assume that “the perceptual ‘something’ is always in the middle of something else, it always forms part of a ‘field’” (Merleau-Ponty, 1945), this cannot apply only to the object which is usually perceived as the main object. It must also apply to other objects and even to the background. To assume that whatever “thing” we see must be in the middle of something else is to assume that what surrounds the thing we see cannot be seen except in the presence of an additional “something else.” Consequently, the outermost region of the perceptual field would not be visible. But we can make this argument not only starting from the center to reach the periphery, but also backward, from the periphery to the center. The regions that we see, like the main background, would *also* have the function of making us see and not of being seen.

The hypothesis that the role of the space surrounding the content is to allow perception is consistent with a phenomenal characteristic of the background. While the characteristics of figure and background are well known, it is not sufficiently emphasized that their phenomenal relationship is not one of mere contiguity or *co-occurrence* in the field. Object and ground are closely interdependent, not only because they are foreground and background, respectively. We know that the background is formless and that it is perceived as empty space (Kanizsa, 1980). This description neglects the fact that the background appears in relation to the object and seems to help give it form, pop-out and phenomenal “matter.” We cannot simply say that the background is less salient than the object. A secondary object or detail is also less salient, but it does not have the same relationship that the background has with the main figure.

If we try to see the background by focusing our attention on it, it is difficult for us to do so, especially near the object, as we are led, somewhat “pushed,” to see the object. Even when we strive to see it as an object, the background still tends to *make us see* the figure it bounds and to make it pop out perceptually. This also means that the background, especially near the object, is phenomenally characterized as a region from which visibility is subtracted. This is why I call this phenomenon *subtraction of visibility*. Naturally, I am referring to a partial subtraction of visibility. The “objective” datum, for which a contrasting surrounding space is necessary for the content to be perceived, is thus consistent with the subjective datum, since a phenomenal property of the background appears to be that of allowing the content to be perceived.

However, the subtraction of visibility is *perceptible* only if this space is bounded by an additional space. About the background, Kanizsa (1980) states that “from a perceptual point of view there are considerable functional differences between the region of the field that takes on the character of figure and the one which plays the role of background. The figure has an object character, it is a ʻthing,’ whereas for the background this character is much less marked, until it is *almost completely* absent when the background is experienced as empty space.” The presence of a residual object character is thus essential to be able to speak of background as an empty space. One can speak of space *in the absolute sense of the term*, and not of background, when the object character is missing *altogether*.

In my view, the loss of the residual object character of a region of the phenomenal field that serves as the background results from the absence of an additional contrasting space bounding it. This loss implies that that space cannot be perceived – not even as empty space. If the space that bounds a content is not bounded by another space – i.e., if it is not also a content – it is not phenomenally defined and, as a result, it is not perceived. The wall surrounding the painting, that we perceive, is also a content. If it is the outermost phenomenal background, it is seen thanks to an additional external space which we cannot see.

When we speak of perceptibility, we usually refer to an *internal region* of the perceptual field, bounded by a contrasting surrounding region. In the periphery of the visual field, where there is a progressive decline of perceptibility and the space component becomes gradually predominant, our ability to define not only objects, but also the spaces to which they belong, progressively diminishes, with no possibility of defining the boundaries of the field. If we consider the outermost region of the perceptual field, we must assume that, while it is necessary to perceive the region it bounds, it cannot be perceived because it is not bounded by another space. This means that the outermost *background* of the visual field, which we barely see and which seems to fade into nothingness without being bounded by any region, is actually bounded by an additional external space which we cannot see, but which somehow makes us see it. It is worth specifying that the outermost background of the visual field is a phenomenal entity and that it is formed by the overlapping of a primary content and a primary space that prevails over the former. The outermost region of the field is not a phenomenal entity, because it is exclusively made up of an absolute space. The outermost region as an additional external space which we cannot see allows us to see the outermost phenomenal background and avoids the endless regression which would occur if we assumed that every background must be surrounded by another - phenomenal - background.

The existence of the subtraction of visibility implies that primary space, while unseen, is not phenomenologically inert. Just as we can say that a primary content – for the way it affects our experience – is phenomenologically positive, we could say that a primary space is *phenomenologically negative*. This characteristic impacts the background through a partial subtraction of visibility. As we have seen above, the phenomenal nature of the background derives from the overlapping of the content and space components when the latter is prevalent. Being phenomenally negative, primary space partially takes away visibility from the region perceived thanks to the surrounding space. This action produces the phenomenal quality typical of the background. Of course, this does not occur only in the background, but in it the phenomenon is more evident. This is a counterintuitive concept, if not contradictory to our idea of the phenomenal world. However, it is interesting to note that, drawing on Gestalt theories, the concept of negative space is used in graphic design and photography. In art and design, negative space is the empty space around and between the subjects of an image (Cave, 2013).

The above is an analysis of the qualitative aspects of early perception. In this paper I am not addressing qualities usually attributable to qualia. I am not explaining the redness of red, but I limit myself to stating that it is something less simple than the quality related to the perception of the phenomenal object. However, the characteristics of the object are no less qualitative than the redness of red and the painfulness of pain.

## Discussion

An approach that has made significant contributions to the understanding of conscious perception is experimental phenomenology, i.e., the study of appearances in subjective awareness (Albertazzi, 2019, 2021; Albertazzi et al., 2021). It aims to uncover the principles of organization that guarantee (qualitative) invariants. These phenomena are explicable on the basis of the conditions of their appearance that the phenomenological analysis is able to demonstrate (Kanizsa, 1979). The fact that in the phenomenological experiments there is a manipulation also of physical stimuli is largely irrelevant because the description, manipulation, and demonstration are performed at the level of appearances only (Musatti, 1957). The kind of information that experimental phenomenology uses to perform suitable behavior in conscious perceiving is internally directly given in present awareness, qualitative in nature. My approach partly distances itself from experimental phenomenology, because MHS is correlated with the SC gradient of the proximal stimulus (Forti, 2015). In other words, I have adopted a psychophysical approach (Gescheider, 1997; Fetsch et al., 2013).

Unlike Gestalt laws, MHS guarantees the unity of perceptual organization. It explains not only how we define the main object, but also the relationships between the parts. By posing the problem of the composite nature of apparently homogeneous regions of the field, it is a necessary premise for explaining the phenomenal and qualitative nature of the different components of the perceptual field. This explanation is made possible by assuming the existence of a hidden conscious structure. The qualities of early vision result from the overlapping of appearing and making something appear and from the relationships of surroundedness between the regions that overlap. These relationships also entail a progressive segmentation of the field, which ensures the unity of perceptual experience.

The parts that cannot be perceived affect all aspects of our experience. The primary perceptual space is essential not only – in an absolute sense – to enable us to see, but also to make us see how we see what we see. Early visual experience corresponds to a Hierarchy of Spatial Belongings. This structure, though hidden from experience, appears consistent with the nature of field parts like object, background, and detail. It is also consistent with the way these parts tend to form a unified whole. In other terms, the existence of a HSB explains why in early perception field components have a certain quality and appear as a unitary set of interdependent components, i.e., the reason why the explanandum consists of a unitary set of phenomenal qualities. Several phenomenal qualities can be traced back to just two factors: (1) the relationship between primary content and primary space in Spatial Belonging, and (2) the existence, in the field of consciousness, of a Hierarchy of Spatial Belongings nested within each other. Even if I do not explain how appearance is defined by the relationship between primary content and primary space, this relationship allows us to provide a relatively parsimonious explanation to the different primary qualities and their interdependence (Schurger and Graziano, 2022).

Moreover, the existence of a hidden conscious structure leads us to change our conception of consciousness. The definition of consciousness cannot be based *only* on appearance or a part of it. Consciousness is not only what appears or what we are aware of. On the contrary, it is also made up of non-apparent or non-perceptible parts, in relation to which we cannot make any phenomenal distinction. We are not aware of all that is part of the conscious field, not only because of the existence of unfocused, fringe or progressively fading parts, i.e., as a result of limited capacity (Zeman, 2001). We are not aware because what appears requires something to make it appear, which in itself does not have the property of appearing, even if the phenomenally negative nature of primary space is somehow made manifest at the phenomenal level as subtraction of visibility. I am referring to fundamental components of the conscious field that are an integral part of its structure and that, being related to the apparent ones, are essential for consciousness to appear as such.

The different nature of content and space makes us understand why, although the hierarchical organization of spatial belongings is compatible with the phenomenal datum, we do not see consciousness in this way. This organization can be considered a kind of hidden architecture of the phenomenal field. The hidden structure of consciousness is explained on the basis of the need to make the content appear and not on the basis of the generic idea that a cognitive system is incapable of examining its own structure (Loorits, 2014).

According to some authors, consciousness is deceptive, either in whole or in part (Dennett, 1991, 2015; Noë et al., 2000). We seem to see more than we see. My analysis also leads to the conclusion that consciousness is, to some extent, deceptive. But my conclusion is quite the opposite, in that I claim that consciousness includes not only aspects which we perceive with difficulty, but also aspects that we cannot perceive.

A lot of authors do not even consider in its entirety what is sufficiently distinguishable. Definitions for which consciousness corresponds to a certain qualitative sensation, or to what it is like to be in a certain state, are based on *a part* of experience, leaving out the parts that are considered non-specific, precisely because they are structural. Other authors neglect the parts that cannot be clearly defined, and they theorize that they need to be eliminated from a scientifically acceptable conception of consciousness. In this way, they hope to simplify the object of investigation. But it is as if we wanted to define the cell by considering the nucleus or the phospholipid bilayer and ignoring everything else.

In my approach, consciousness is deceptive not because we think we perceive more than what we actually perceive. On the contrary, it is deceptive because we do not perceive parts that should be considered to all intents and purposes as belonging to the field and that play a major role in defining the phenomenal quality we perceive. At the same time, the existence of non-perceptible parts is entirely compatible with what we perceive, with our experience. In no way does my analysis lead to overturning the fundamental assumption that, in the case of consciousness, appearance is reality (Chalmers, 1995; Searle, 1997; Tononi and Koch, 2015; Whiting, 2016; Merlo, 2020), nor does it lead to questioning its existence (Dennett, 1991). However, consciousness is not *just* appearance.

The presence of non-apparent, even phenomenally negative components implies that consciousness is much more complex and internally structured than we think. The complexity of experience is usually underestimated by seeking it outside of experience. It is generally assumed that, for consciousness to arise, particularly complex processes occur within or outside the classical canons of neuronal architecture (Tononi and Edelman, 1998; Sarasso et al., 2021; Koculak and Wierzchoń, 2022; MacIver, 2022; Hunt and Jones, 2023), or even physics (Hameroff and Penrose, 2014; Zhi and Xiu, 2023), at the non-conscious level. In contrast, little is said about the complexity of a conscious image that we experience, except for the insights provided by the phenomenological approach (Kanizsa, 1979; Gallagher and Zahavi, 2008; Smith, 2018). However, we have seen that all qualities – even the simplest one, related to the object – result not only from relations with juxtaposed regions, but also from overlapping between different field regions.

The phenomenally negative nature of space makes it possible to perceive the spaces surrounding the objects. This results in an “aerial” structure of experience. Indeed, the experience is made up of material objects located in a space which, while appearing phenomenally incorporeal, is in fact part of the experience. It is worth emphasizing the adaptive value of this kind of structure, which somehow reproduces a world made up of objects and regions in space. The salience of the object corresponds to a consistency that testifies to the material nature of the object and contrasts with the incorporeality of the surrounding space. Despite the similarities, this hypothesis is different from that of Jerath and Beveridge (2019), according to which a subconscious, virtual, space–time matrix is the foundation of experience and continuously exists in the conscious mind as a coordinate system for a recreation or simulation of the material world.

To accept this hypothesis is to accept that from the very beginning PC is made up of components that are not only juxtaposed, but also overlapping. In some cases, the composite nature of our experience is quite clear, although its importance is rarely emphasized: some examples are the scent of a flower, the color of a triangle, the voice of a person, the name of an object. Dennett (1991) uses an example of learning to hear fine details of a guitar sound. Guitar sound can be decomposed into overtones, or constituent parts of the sound. In fact, due to overlapping, the structure of consciousness is composite, complex, and counterintuitive. Being phenomenally negative, space allows us to postulate the existence of far more overlapping sub-images than we imagine. However, even in cases where the components are sub-phenomenal, the nature of the combinatorial effect is not very different. The nature of the background seems to derive from the overlapping of the simultaneously space-like and object-like nature of the region surrounding the main object, in a manner not unlike how the nature of a yellow triangle seems to derive from the overlapping of shape and color.

In conclusion, in order to understand how consciousness is made, we have to break it down into the hidden component and the manifest component of which each Spatial Belonging is made. Then, we have to take into account that what we see comes from the “assembly” of the individual Spatial Belongings in a hierarchical structure. It could be argued that this is an assembly of brain components. However, the individual Spatial Belongings correspond to the realization of the property that is needed, at a sub-phenomenal level, to be able to speak of a minimal state of consciousness, i.e., the appearance which, at the phenomenal level, manifests itself in the possibility of perceiving and distinguishing each conscious content.

The hypothesis of a conscious state having components that we cannot experience seems counterintuitive, or even contradictory. However, if sufficiently well-founded insofar as it is compatible with PC, we cannot rule out this hypothesis. Such a structure may provide a kind of link that can bridge – or at least reduce – the explanatory gap between experience and brain processes and thus help solve the hard problem. With their hidden component, appearance-related processes can account for more complex and differentiated aspects, such as phenomenal and qualitative aspects, on the basis of a few simple principles. In this paper, I only analyze those related to early perceptual experience.

At the same time, the hidden structure of consciousness may be more easily explained in terms of brain organization. Phenomenal experience and brain structure are too different or “distant” to be directly compatible. If we think of the difference as a kind of excessive gap, which would imply *direct* non-reducibility, there is an alternative possibility to the dichotomy between dualism and monism. It consists in hypothesizing that the structure of consciousness, while not conscious in the full meaning of the term – and thus equatable to phenomenal experience – nor directly accessible to introspection, is also not equatable to a non-conscious state. The hidden structure of consciousness, which I have identified in HSB, can be considered a bridge structure which places itself at an intermediate level between experience and physical properties.

As stated by Loorits (2014), it is the non-structural nature of qualia that makes them extremely difficult to explain at the brain level. In this model, the qualities of early visual experience correspond to the HSB. The structural nature of the HSB makes it more easily explicable in terms of brain organization, helping to bridge the explanatory gap between physical properties and experience. Although the limitations of the paper do not allow us to address this question, I will briefly mention a possible direction of research.

An important aspect to take into consideration is that the structure of the HSB is unitary. All interactions involving individual Spatial Belongings and their organization on different hierarchical levels are simultaneous, closely integrated and involve the whole field. Therefore, it is likely that this structure cannot be provided by conventional neuronal organization. Most of neurobiological theories of consciousness look primarily to synaptic firing as the physical substrate of consciousness. However, all neurons also produce electromagnetic fields. Various spatiotemporal scales of electromagnetic fields are generated by, but not identical with the anatomy of the brain. Jones and Hunt (2023) “suggest that these fields, in both their local and global forms, may be the primary seat of consciousness, working as a gestalt with synaptic firing and other aspects of neuroanatomy to produce the marvelous complexity of minds.”

Field theories have made real progress in explaining how fields integrate colors to form unified pictorial images (Jones and Hunt, 2023). At the same time, these hypotheses do not seem capable of explaining qualia. However, the compatibility of electromagnetic fields with the HSB, which in turn is compatible with the qualities of early vision, could be explored. If we refer to early vision, these fields could be supported by brain areas whose units are linked by a grid-like connectivity (Haun and Tononi, 2019). Some of them are retinotopic maps that retain, at least approximately, the relationships present in the field of the proximal stimulus. But, of course, several other hypotheses can be considered.

A significant limitation of this model is that the HSB does not explain consciousness outside of early visual experience. Other phenomenal aspects like feelings, emotion, imagination or dreaming need to be addressed. As I have argued, their explanation probably requires a higher structural level.

What I have proposed in this paper is a possible explanation of what I have identified as the explanandum, i.e., the unitary set of qualities we find in early vision (Forti, 2024). It is precisely elements of that explanandum that help provide the explanation. In turn, the explanation, which consists in postulating the existence of a Hierarchy of Spatial Belongings nested within each other, is an additional explanandum. Exploring the nature of this explanandum requires further research.

## Data availability statement

The original contributions presented in the study are included in the article/supplementary material, further inquiries can be directed to the corresponding author.

## Author contributions

BF: Writing – original draft.

## Footnotes

^1^Of course, the background of a sound can also be temporal. For the sake of simplicity, here I limit myself to spatial backgrounds, both by analogy with vision and because spatial relationships do not require the involvement of memory.

^2^In order not to generate confusion, I use the term overlapping also in cases in which the term superimposition might be more appropriate.
